# How to Test Chloroform

**Published:** 1880-04

**Authors:** 


### HOW TO TEST CHLOROFORM.

The chemist, Regnauld, gives these directions for testing chloroform: 1. Pour some of it on bibulous paper folded several times, and when nearly all evaporated, observe the odor. Pure chloroform retains its mild pleasant smell to the very last, leaving the paper dry and odorless; whereas, an impure article emits a disagreeable odor, partly nauseating, partly acrid, while the paper remains somewhat moist. Chloroform of this later description must be rejected for inhaling. 2. Free hydrochloric acid is indicated by turbidity caused by silver nitrate, or when litmus paper is turned red. But in the presence of uncombined chlorine the test paper becomes decolorized. 3. Into a test tube place some potassium hydrate and a few drops of water. Then add one or two ccm. of chloroform and heat to ebullition. A yellow or brown coloration indicates aldehyde. 4. Shake together in a test tube, equal volumes of chloroform and colorless concentrated sulphuric acid, and allow the liquids to separate. They should remain colorless. If they assume a brown or brownish-red hue, chlorinated derivatives of propylic, butylic and amylic alcohols are probably present. 5. The boiling point of pure chloroform is 60.8° C., at a barometric pressure of 0.760.

When the liquid boils below this point, sulphuric ether is present; and if later on it exceeds the point, it is contaminated with other chlorine compounds. 6. C. Remy has recently fixed the specific gravity of pure chloroform at 1.500 at 15° C.

